# The challenges of reshaping disease specific and care oriented community based services towards comprehensive goals: a situation appraisal in the Western Cape Province, South Africa

**DOI:** 10.1186/s12913-015-1109-4

**Published:** 2015-09-30

**Authors:** Helen Schneider, Nikki Schaay, Lilian Dudley, Charlyn Goliath, Tobeka Qukula

**Affiliations:** School of Public Health, University of the Western Cape; MRC/UWC Health Services to Systems Research Unit, Robert Sobukwe Road, Bellville, Cape Town, 7535 South Africa; School of Public Health, University of the Western Cape, Robert Sobukwe Road, Bellville, Cape Town, 7535 South Africa; Division of Community Health, Faculty of Medicine and Health Sciences, Stellenbosch University, Francie van Zijl Drive, Tygerberg, 7505 South Africa; Division of Community Health, Faculty of Medicine and Health Sciences, Stellenbosch University & Department of Health, Western Cape Government, 4 Dorp Street, Cape Town, 8000 South Africa; Community Based Services, Department of Health, Western Cape Government, 4 Dorp Street, Cape Town, 8000 South Africa

**Keywords:** Community health workers, Lay health workers, Community based services, Community system strengthening, Situation appraisal, Governance, South Africa

## Abstract

**Background:**

Similar to other countries in the region, South Africa is currently reorienting a loosely structured and highly diverse community care system that evolved around HIV and TB, into a formalized, comprehensive and integrated primary health care outreach programme, based on community health workers (CHWs). While the difficulties of establishing national CHW programmes are well described, the reshaping of disease specific and care oriented community services, based outside the formal health system, poses particular challenges. This paper is an in-depth case study of the challenges of implementing reforms to community based services (CBS) in one province of South Africa.

**Methods:**

A multi-method situation appraisal of CBS in the Western Cape Province was conducted over eight months in close collaboration with provincial stakeholders. The appraisal mapped the roles and service delivery, human resource, financing and governance arrangements of an extensive non-governmental organisation (NGO) contracted and CHW based service delivery infrastructure that emerged over 15–20 years in this province. It also gathered the perspectives of a wide range of actors – including communities, users, NGOs, PHC providers and managers - on the current state and future visions of CBS.

**Results:**

While there was wide support for new approaches to CBS, there are a number of challenges to achieving this. Although largely government funded, the community based delivery platform remains marginal to the formal public primary health care (PHC) and district health systems. CHW roles evolved from a system of home based care and are limited in scope. There is a high turnover of cadres, and support systems (supervision, monitoring, financing, training), coordination between CHWs, NGOs and PHC facilities, and sub-district capacity for planning and management of CBS are all poorly developed.

**Conclusions:**

Reorienting community based services that have their origins in care responses to HIV and TB presents an inter-related set of resource mobilisation, system design and governance challenges. These include not only formalising community based teams themselves, but also the forging of new roles, relationships and mind-sets within the primary health care system, and creating greater capacity for contracting and engaging a plural set of actors - government, NGO and community - at district and sub-district level.

**Electronic supplementary material:**

The online version of this article (doi:10.1186/s12913-015-1109-4) contains supplementary material, which is available to authorized users.

## Background and rationale

South Africa shares with the rest of southern Africa the presence of a large community based health sector engaged in a wide variety of care, support and advocacy activities [1]. Initially heavily focused on community mobilisations around, and receiving funding for, HIV/AIDS [2], the last few years have seen moves in the region towards greater integration of community based programmes within health systems, and in particular, the formalisation of national community health worker (CHW) programmes [3].

This reflects a global pendulum swing back towards CHWs as a recognised cadre in national health systems. At a special session of the Third Global Forum on Human Resources for Health in Brazil in November 2013, a gathering of key global actors reaffirmed the significant role that CHWs could play in accelerating achievement of the millennium development goals (MDGs) and Universal Health Coverage. It called for the strengthening of CHW programmes and their integration into national health systems [4].

In line with these developments, South Africa is reorienting a loosely structured and highly diverse community care system that evolved around HIV and TB, into a formalized, comprehensive and integrated primary health care (PHC) outreach programme, based on CHWs. A government audit in 2011 counted more than 72,000 facility and community based lay health workers linked to health departments across the country. While heavily funded by government, these workers have been employed and stipended through nearly 3,000 community based organizations [5]. The new proposals, referred to “PHC Re-engineering”, envisage a reorganization of this community based care infrastructure into “PHC Outreach Teams” of CHWs, led by professional nurses and ultimately absorbed into the government staff establishment. The outreach teams will be responsible for a defined number of households and will be accountable to the local health facility. Their roles will be comprehensive: extending beyond HIV/TB to include maternal-child health and chronic non-communicable diseases; they will have a preventive and promotive orientation, and with other sectors and community based providers, will address social determinants of health [6]. PHC Re-engineering is itself located within a broader set of reforms under the umbrella of Universal Health Coverage (referred to as National Health Insurance) in South Africa.

The reorientation and strengthening of an existing, fairly extensive, government supported infrastructure offers many opportunities, but also constraints in the already established status, roles, management systems and governance arrangements of community based services. The strengths and weaknesses and historically shaped nature of existing systems have to be understood when implementing new policies. While each context is unique, the case of South Africa may offer general lessons for other countries undertaking similar reforms.

This paper examines the implications of the new policy direction of PHC Re-engineering at a sub-national level in South Africa. While a national mandate, provinces have a fair degree of autonomy in adopting and adapting national policy, especially if they are required to mobilise the funding for implementation. The paper thus reports on the findings of an appraisal of community based health services in the Western Cape Province, commissioned by the health department as it was formulating a long term provincial strategy referred to as Healthcare 2030 [7]. Healthcare 2030, developed by provincial policy makers and managers, prioritises strengthening and expansion of community based services in line with national PHC Re-engineering, proposing new norms for availability of community health workers, mid-level cadres and supervisory professionals. It emphasizes an approach focused on prevention, promotion and tackling the social determinants of health, while retaining a *“complementary capacity for curative, rehabilitative and palliative care”* [7]. A notable feature of the Healthcare 2030 Strategy is its emphasis on values and principles: key concepts are person-centredness, continuity and integrated provision, participation, primary health care, district health systems and an outcome-oriented approach. Table 1 below summarises the key policy recommendations envisaged for community based services in Healthcare 2030.

**Table 1:** Policy recommendations for community based services in Healthcare 2030

Policy dimension	Policy recommendation
Roles	Comprehensive orientation including preventive, promotive, care and rehabilitation;
	Community based action on determinants of health as part of a broader inter-sectoral focus on wellness;
	Outcome oriented approach focused on major causes of ill-health in the province: HIV/AIDS and TB, chronic non communicable diseases, violence and injury, mental health, maternal (parent) infant and child health, early childhood development;
Target population	Population based model in which teams are responsible for the health of a defined population (electoral wards in urban/metro areas, sub-district rural areas);
	Proactive approach to all households;
Links to health care system	Integral part of public primary health care system, supervised and supported by facility based staff;
Team structure and ratios	Each CHW works 8 hours a day and responsible for 270 households;
	Team of 10 CHWs to be supported by one Clinical Nurse Practitioner;
	One rehabilitation care worker per 8 CHWs;
CHW training	Core roles and training standardised, based on a nationally accredited curriculum;
M&E system	Standardised M&E systems reporting on key indicators;
	Use of mHealth strategies for M&E;
Value system	Person/patient centred;
	Community embeddedness: stable, long term relationships with households which build empathy and trust.

There is considerable international literature on the roles and efficacy of CHWs [8, 9], and to some extent on immediate support systems (e.g. supervision and incentives) required [10], but little evidence on the management of CHW programmes at scale through national health systems [11]. McCord, Liu, & Singh [12] propose that CHW programmes be viewed fully as a sub-system of the health system, embedded within primary health care. Using the WHO *“building blocks”* framework of a health system [13], they outline the elements of this sub-system: service delivery, workforce, information systems, supply systems, finance and leadership and governance. Similarly, in a wide ranging guide on strengthening CHW programmes at scale, Perry & Crigler [14] assert the need for embedding CHW programmes within national and sub-national system contexts, and for planning based on situation appraisals that assess such contexts. They go further than the building blocks approach in emphasizing the relational dimensions of CHW programmes – both in relation to the formal health system and communities.

This paper aims to contribute empirical evidence on the challenges associated with reorienting community based services, which have their origins in disease specific responses to HIV/AIDS and TB, towards the new processes and goals outlined in Table 1. Using a systems perspective that focuses both on the structure (the “hardware”) and actor mind-sets and relationships (“software”) [15] of CBS, it appraises the current system with the view to highlighting the key dilemmas and challenges faced by health system stewards in reshaping community based services in new ways.

## Methods

Over a period of eight months (November 2012-June 2013), a team of nine researchers, working in close collaboration with provincial stakeholders, conducted an in-depth appraisal of community based services (CBS) in the Western Cape Province. Western Cape is one of nine provinces in South Africa and has a population of 5.8 million. The health sector is divided into 6 districts (5 rural and one metro) and 32 sub-districts. The sub-district (or sub-structure as it is referred to in the Metro) is the most decentralised level of governance in the Western Cape’s health system, and corresponds in size and function to the classic WHO concept of the District Health System, encompassing community based, primary health care and district hospital services.

**Fig. 1:**
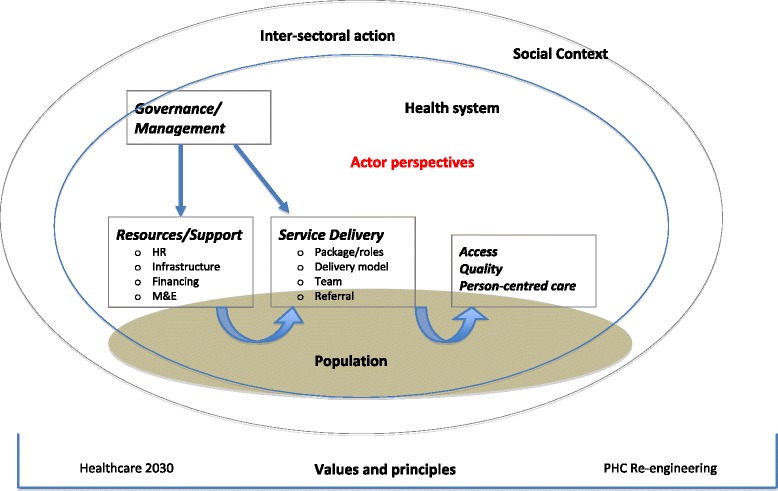
Conceptual Framework adopted in the appraisal

Data collection and analysis were guided by a health system framework (adapted from van Olmen et al. [16], assessing outputs (e.g. access, quality), the organisation of service delivery (e.g. roles, supervision), resources and systems (e.g. financing, M&E), and governance/management arrangements (e.g. NGO contracting, accountability relationships) (Fig. 1). The framework also emphasizes values and principles (as espoused by Healthcare 2030, for example), the social and inter-sectoral context in which CBS is embedded, and the health system as interacting with populations.

In addition to mapping these dimensions we sought to understand system strengths and weaknesses through the eyes of the actors in and around CBS, and specifically how they saw its potential and future. The appraisal methods thus included semi-structured interviews or focus group discussions with a cross section of stakeholders from senior to frontline, including health facility managers, providers, patients, community members and stakeholders from other sectors (e.g. social development); analysis of routine data; observations of community care worker (the term for CHWs in the province) practice; and document reviews. Two rural and one urban sub-district/s, were selected for in-depth study by the provincial government as representative of the two realities. The rural sub-districts formed part of a national pilot for national health insurance reforms, and the urban sub-district contained the range of settlements and service responses available within the province.

A total of 97 key informant interviews, 10 focus groups discussions, 23 observations of community care workers (hereafter referred to as CHWs) and 16 patient “care pathway” interviews were completed (Table 2). Key informants and focus group participants were purposively selected, to represent the range of players providing, managing or receiving CBS. Interviews were conducted in the preferred language of the participants, in their work and home settings and in private.

**Table 2:** Summary of data collection activities at a provincial and local levels

Area	Key informant interviews	Focus group discussions		CHW	Patient interviews
				Observations	
		Community	CHW		
Rural sub-districts	50	3	4	16	14
Urban sub-district	24	2	1	7	2
Provincial	23				
Total	97	5	5	23	16

Following the framework, the content of the interviews covered the various dimensions of the community sub-system as (emphasising aspects most relevant to each actor) but also assessed the acceptability of the Healthcare 2030 strategy and readiness for change towards the new approach. Amongst the key informants were the managers of 14 NGOs, 12 of whom provided information on the types/categories of CHWs in their employ (n = 409). In addition, the provincial Community Based Services Directorate made available a database with information (age, sex, education, duration in employment, and training) on 2,893 CHWs across the province. Focus group discussions with community members focused on the knowledge, role and acceptability of community based services. The observations involved a researcher accompanying a CHW, purposefully selected to represent the range of cadres and geographical realities in the province, over a day’s work (starting at their homes), and were guided by an observation checklist. Patient interviews examined the care pathways of individual patients from the start of the illness to the present, exploring the role and perceptions of community based services in this pathway. The study proposal was assessed and approved by the University of Stellenbosch’s Ethics Committee, and informed consent and guarantees of confidentiality preceded all interviews.

Interviews and focus group discussions were recorded and transcribed and notes of observations written. Routine data were obtained in excel format and entered into Stata (Version 12) for further analysis. Data analysis was conducted iteratively over a number of weeks, in which responsibility for producing preliminary reports was divided amongst team members, followed by extensive discussion and triangulation of data sources. The health systems framework provided a structure for describing the state of CBS (in a deductive manner), while a significant part of the final report was devoted to an inductive thematic analysis of stakeholder perspectives in which we reported both on current key concerns and visions for the future. Three separate report back workshops were held, for the purposes of both member checking and generation of recommendations. These then formed the basis of a final appraisal report presented to a meeting of senior managers of the provincial health department [17].

## Results

Additional file 1: Table S1 summarises the findings of the appraisal following the categories of the system framework, as well as the challenges associated with and recommendations for shifting the current community based services platform towards the goals of Healthcare 2030 as outlined in Table 1. Key themes are explored further below.

### CBS delivery model

The current CBS delivery model dates back to the emergence of community based responses to HIV/AIDS and the related epidemic of TB in the mid to late 1990’s. In 2003, European Union (EU) funding, aimed at promoting the development of community based organisations, enabled the expansion of home based care across the province through a system of NGO contracting. The initial focus was on “dehospitalised” and palliative care for bedridden patients, in an era when anti-retroviral therapy (ART) for HIV was not yet universally accessible. Simultaneously, NGOs in the province were experimenting with community based models of TB care, based on the WHO “DOTS” – directly observed treatment, short course – approach. When the EU programme ended in 2007, a combination of funding from an Expanded Public Works Programme (EPWP) and national ring fenced grants for HIV and TB enabled the continuation and expansion of this service platform. With greater access to ART, the focus of home based care shifted to dehospitalised care of other chronic diseases, adherence support for those on ART and TB treatment, and most recently, school health services. Initially NGO-based services were single purpose (TB, HIV, nutrition, palliative care etc.) with models and approaches to delivery specific to each NGO. Through the contracting process, the province has sought to incrementally shift service delivery to more integrated roles (e.g. combining TB and ARV adherence support), specifying standardised core packages and demarcating geographical zones of NGO activity.

At the time of the appraisal (mid-2013), the Western Cape had a well-established CBS delivery platform provided through contracts with 72 NGO intermediaries, and employing 3,594 CHWs. This represented a ratio of 0.78 CHWs/1,000 public sector dependent population, a fairly extensive infrastructure, but still significantly less (shortfall of 28 %) than the new norms proposed by Healthcare 2030.

The vast majority (97 %) of CHWs were women, typically between 30 and 50 years of age; all had some secondary level schooling (40 % with a school leaving certificate). They worked half days, earning stipends of R1,200-R1,500 ($US120-50) per month. They were recruited by the NGOs and supervised by nurses in a ratio of roughly 20 CHWs to one nurse supervisor. This is in contrast to the full time, implicitly better remunerated, and better supervised worker proposed in Healthcare 2030. The numbers of CHWs, nurse supervisors and stipend levels were determined by the provincial government and specified in NGO contracts. While initially managed by a provincial CBS Directorate, NGO selection, contracting, disbursement and financial accounting was being decentralised to district structures at the time of the appraisal.

### Roles of CHWs

The majority of the 409 CHWs inventoried in the two sub-districts (73 %; urban, 66 %; rural, 96 %) were referred to as *“home based carers”*, who described their roles principally as providing support for activities of daily living (washing, feeding, changing bedding), basic nursing care (wound dressing, pressure ulcer care, disposal of needles and syringes), limited rehabilitation (walking, sitting), and general emotional support in homes. The patients were referred for home based care to the NGO by local hospitals via district/sub-district channels.

The second most common role was that of *“CDL - chronic disease of lifestyle – worker”* provided by a single purpose cadre in urban areas and by home based carers in the rural sub-district. This role involved running “CDL clubs” (support groups of patients predominantly suffering from hypertension and diabetes), and where nursing professionals were available, the distribution of follow up chronic disease medication. In this instance, referral and support relationships were established with local health facilities.

The third role was that of ARV/TB treatment adherence supporter (also a single purpose cadre in urban areas), which in contrast to the CDL clubs, followed up clients in their homes, doing pill counts, treatment literacy, tracing of contacts and defaulters, and liaising closely with local TB/HIV clinic staff.

Finally, in a recent addition to services, CHWs were deployed to school health teams to assist nurses with health promotion and screening programmes, most especially in the urban district.

While there appeared to be a general convergence of CHW roles towards these core activities, a few government supported NGOs had retained distinct identities and roles, which included, amongst others, outreach and support (nutrition, breastfeeding, integrated management of childhood illness etc.) to pregnant women and young children, and holistic (combining health and welfare) family support to households at risk, identified through house to house visits.

As the time of the appraisal, the profile of roles was far from the comprehensive vision of Healthcare 2030. The majority of CHWs and activities were oriented towards provision of home nursing care of patients referred to NGOs following discharge from hospital, and community based follow up/support for chronic life long conditions. Roles and activities focused on children, reproductive health and young adults were largely absent. Preventive and promotive roles were limited to periodic community campaigns (organised along “seasons”).

In observations of CHWs their roles, particularly in households, were often vague and lacking in definition, tending to follow a limited number of locally mandated routines, and heavily focused on meeting daily visit quotas. Patient journeys and observations documented large numbers of missed opportunities for intervention (in all age groups) within households. A significant part of the day, especially in rural areas, was consumed with walking to and from the homes of patients.

Many of the district actors questioned the value and quality of services provided by NGOs and CHWs: *“We have no idea what they are really doing, we don’t know what the quality is of the work they are doing*.“(District Manager) Roles had also evolved in an ad hoc manner. *“CBS takes on new programmes each year in an unstructured fashion and without a clear plan.”* (Sub-district CBS Manager). Stakeholders from other sectors commented on the absence of collaborative efforts with similar workers in related sectors, such as social development.

In our observations, CHWs were also treated as subordinate cadres. They followed the instructions of professionals and were readily drawn into facilities to undertake menial activities. Interviewees pointed out that CHWs were very familiar with their neighbourhoods and *“know exactly who has given birth, who is a drug addict…who has an emotional (problem).”* (NGO manager), yet the nurses and other professionals they interacted with seldom asked them to contribute insight or opinion on clients or households. They did not appear to be seen as agents with independent knowledge of community life and capable of judgement and discretionary action.

Stakeholders across the board were in favour of a revised definition of roles for CHWs in line with Healthcare 2030 and believed that CBS held considerable potential for addressing disease burdens. Amongst senior managers there was near universal support for a comprehensively trained community health worker, with a stronger preventive and promotive focus, more strongly embedded within the PHC system and working with other sectors. *“There is very little being done with health promotion. That is where we are going to save resources and build a healthier nation if we focus on promotion. We need to bring in other partners. Therefore this cadre of staff needs support from other sectors as well in order to do health promotion…”* (Provincial Manager) *“[We] need to have a generic worker, a ward based system, good supervision, good links to PHC and a good M&E system”.* (District Manager)

Stakeholders also reaffirmed the role of CHWs as complementing that of local health facilities and the need to strengthen links between PHC facilities and communities: *“It’s not really about treatment it’s about education, training, screening…. facilities are there to treat and [CHWs] are there to promote and prevent…”* (Provincial Manager) *“They are supposed to strengthen the system from the clinic to the community, and from the community back to the clinic.”* (District Manager)

Community members also welcomed the possibility of new roles, provided there was sufficient preparation and participation: *“Communities should be part of the safety system and they can play a big role in if they were educated about the new vision and have knowledge about the new system.”* (Community FGD discussion). Acceptance of and support for CHWs was universally high, and the simple act of visiting people with illnesses in homes was valued. CHWs were contrasted favourably with health professionals: *“Indeed they [CHWs] are a great help, they understand your environment more than the ones [nurses] that are in the clinic because they come to your homes and see your condition, that in itself helps you as a person.”* (Community focus group discussion). This trust in CHWs suggests a degree of community embeddedness and the potential role as mediator between communities and the health system.

At the same time, there was uncertainty as to what roles would lead to the most effective outcomes. As one district manager pointed out: *“The problem is that it is such a wide concept [community based service delivery], and each person interprets the concept in their own way… [they are] all on different pages. It’s a very broad concept. [I] don’t think management understands it or is fully in agreement on what it should be.”* (District Manager)

The was also *“a fear that we are going to overburden these people [the CHWs]. They don’t have the hands.”* (Provincial Manager), while others pointed to the need for local prioritisation: *“The burden of disease in particular areas should dictate this [the role], some areas have different health issues.”* (Provincial Manager)

One NGO was concerned that the new focus on prevention and promotion would be at the cost of providing home-based care for those that are already ill: *“This could make a great impact - but only if they don't lose focus on those who are actually ill, and not just (focus) on prevention and promotion activities. It should be prevention, promotion and care.”* (NGO manager)

### Human resource dimensions

A quarter (26 %) of CHWs had been working for five years or more, while 48 % had been working for less than two years, representing a high turnover around a stable core. In the NGO inventories conducted in the urban sub-district, 31 % of CHWs had left their organisation in the year prior to the appraisal. The low stipend and precarious nature of employment were seen as the main reasons for this: *“Where there are no benefits, no job security or a fulltime job, there will be a high turnover of carers.”* (Sub-District Manager)

A key consequence of the high turnover was that just over half (51 %) the CHWs were at “entry level”, namely, had not had an opportunity to be trained through the nationally accredited and laddered (from 1 to 4 years) training system developed for community carers. Funded by the national Expanded Public Works Programme and provided through contracted private providers, vigorous attempts were being made by provincial managers to ensure that all CHWs had access to some training. However, training providers and processes were disconnected from NGOs, who resisted sending much needed personnel away for periods of time to be trained. Interviewees across the board saw this training system as poorly aligned to current (let alone future) needs, as expensive, unrealistic (stretching over 4 years) and as failing to secure any meaningful career pathways. Apart from additional training provided by the provincial government for TB/HIV adherence workers, systems of in-service and induction training were the responsibility of the individual NGOs. Skills and therefore quality of services were highly uneven across the platform.

### Financing and M&E systems

Provincial funding streams for CBS have been stabilised through a combination of EPWP and national HIV/AIDS conditional grants. However, these funding sources are outside of the core provincial budget, referred to as the “equitable share”, which funds the primary health care and district health system. Although the management of CBS was being increasingly decentralised, its location as a contracted service funded through special mechanisms reinforced its status as an optional add-on that would survive as long as these mechanisms were available.

Despite the low levels of remuneration and very limited resources for transport, communication and uniforms, and the small proportion of total provincial health expenditure devoted to CBS (estimated to be less than 2 %), senior managers expressed uncertainty about the large amounts of money *“signed off”* in NGO contracts on an annual basis. *“If you ask me for the millions we spend on this programme, what exactly is the outcome of that, I can’t tell you, I have no idea.”* (District Manager) While financial management and NGO governance systems were adequately monitored, NGOs were not being held accountable for their performance. *“…there are NGOs who are performing and those who are not performing. (The Department) cannot just continue to fund for the sake of funding. NGOs need to display that they do actually have disciplinary procedures that they use in order to ensure the quality of their work.”* (NGO Manager)

This concern also related to the perceived absence of monitoring and evaluation systems. “*There is no M & E framework…adequate attention is not paid to the impact and what is being provided there.”* (Provincial Manager) At the time of the appraisal NGOs were in reality returning routine monthly activity reports through an elaborate CBS information system that involved 46 data elements and extensive form filling. This system produced information that was regarded as being of poor quality and which no one trusted. There is difficulty in capturing, in a set of routine indicators, a diffuse and shifting service delivery platform, and CBS has fallen largely outside of quarterly and annual systems of review and reporting, entrenching its marginal status.

Thus while CBS was being seen as holding great potential, there was reluctance to consider allocation of additional resources: *“There are many expectations from CBS but no resources committed to it.”* (Sub-district Manager)

### Governance

A key theme raised during the appraisal was the most appropriate organizational location of community based services and CHWs – as integrated into the public sector workforce or as remaining within the NGO sector. While national proposals were initially for integration into the civil service, where salaries and conditions of service would have been greatly improved, the absence of new budget lines has made this difficult to implement in the short term. Affordability is however, just one of the considerations, and it is possible to envisage an NGO model where CHWs are adequately remunerated. Table 3 summarises the advantages and disadvantages of the NGO and DOH (integrated) models of service delivery raised during the appraisal.

**Table 3:** Advantages and disadvantages of DOH and NGO models of provision

DOH provision		NGO provision	
Advantages	Disadvantages	Advantages	Disadvantages
• financial security	• CHWs easily become facility based	• aligns with NHI contracting models	• variable supervision and capacity
• personal job security	• curative oriented	• more responsive, innovative and efficient	• power dynamic between DOH and NGO unequal
• career paths & promotion	• barriers to entry, some excluded	• community ownership & identity	• CBS may not be their primary activity
• standardisation of roles	• massively increase the costs	• inter-sectoral action more feasible	• funding streams vulnerable in the current financial climate.
• easier to control	• CHWs would lose their community identify	• primary prevention focus	
• better access to resources and supplies		• advocacy for particular issues	
• continuity of care & integration		• history, credibility, & networks	
• better alignment with the DoH outcomes			
• lower transaction costs in managing contracts			
• in-service training is easier			

While many reflected on both the pros and cons of the current system, views on the desirability of NGO-contracted model varied considerably. Senior provincial and district managers were firmly of the view that the NGO model should continue. Positions varied from the principled: *“The NGO model has a lot to offer, let’s figure out how to do it better”*, to the pragmatic *“We’d like to bring them into the system but we’ll never afford it”.* At lower levels, amongst PHC providers and front line managers involved in delivering and managing services there was, however, much stronger criticism of the NGO model. There were effective systems of financial management, but the day-to-day performance of CHWs and NGOs was seen as difficult to manage and control. NGOs had their own (diverse) imperatives and did not necessarily share the vision of the health department; the system was unstable with poor retention and high turnover of CHWs due to low stipend payments; there was little direct supervision of CHWs by NGO nurse coordinators; and referral systems into the platform were complex.

A related theme was the lack of clear lines of coordination, communication, referral and accountability between CHWs, NGOs and local primary health care facilities, stemming in part from the “dehospitalisation” focus of the platform. Many expressed the view that *“…there needs to be closer link between the facility based and community based services so that the facility based services take (the CHWs) seriously enough.”* (CBS manager) While there had been decentralisation of NGO contracting, this went as far as the district level and tended to be associated with HIV/AIDS programmes. Those responsible for the day-to-day management of primary health care and district services, at a sub-district level, had little involvement in the decision making or planning for CBS.

## Discussion

The findings highlight a complex and inter-related set of design, resourcing, relational and governance challenges for provincial managers and policy makers seeking to reorient community based services towards new goals as proposed in Healthcare 2030. In sum, measures are required to ensure greater retention and stability of the existing core cadres while expanding numbers, redefining and extending roles, investing in training, designing new, aligned and integrated management systems, reshaping relationships in the primary health care system between CHWs, NGOs, communities and other sectors, and creating capacity for governance of community based services at local levels. These are discussed further below.

In many respects these challenges are not new and were well described in the first wave of large-scale CHW programmes following the Declaration of Alma Ata in 1978 [18, 19]. However, these old challenges are confronting a new and different context, particularly in the southern African region. The first aspect of this is the highly diverse, organic and dense character of the current community care infrastructure that evolved in response to HIV/AIDS. While this mobilisation offers significant opportunities, it may also not be readily amenable to reshaping as a regulated form of health sector outreach, especially if this is not accompanied by access to significant new resources [20]. The second aspect of this context is the limited scope of existing interventions and the need to negotiate and develop a more comprehensive repertoire of roles and responses, capable of adapting to a complex and changing set of needs.

### Design

In the Western Cape, the first challenge consists in defining the place of CHWs and outreach teams in line with the priorities and approach of Healthcare 2030. Unfortunately, much of the international evidence on CHW roles, especially in Africa, relates to the achievement of the Millennium Development Goals, and most specifically to the maternal and child health aspects of these [8, 9]. The settings of this evidence base are different to the Western Cape where infant and child mortality are relatively low (21 and 25/1000 live births in 2010, respectively), and where chronic conditions (HIV/TB and other non communicable diseases) and violence and injury (underpinned by substance abuse and mental illness) dominate as causes of ill-health and mortality [21].

Beyond defining roles, the location of CBS between the health system, households and community, and of CHWs as having a double identity as representing both [22], also poses more fundamental questions on how best to frame this aspect of health systems. Should community based services and CHW programmes be conceptualised as engaging and mobilizing communities to address the social determinants of health, or as a form of health system outreach with specific technical roles? This distinction, famously characterised as “lackey or liberator” by David Werner in the 1980s [23], while a simplistic representation of fluid and hybrid everyday realities, points to the importance of clarifying underlying assumptions and establishing the basic identity of CHW programmes.

Shifting from a limited purpose care and referral service, oriented to needs of hospitals to a pro-active and comprehensive engagement with households and communities requires a fairly radical reshaping of roles and relationships within the PHC system as a whole. At formal level, the CBS teams proposed in Healthcare 2030 will become much more closely linked to PHC facilities in referral and reporting relationships, in contrast to the diffuse (hospitals, PHC, HIV/TB programme) relationships that characterised community based services in the past. On the other side, a more active and systematic approach towards households can only succeed if there is a degree of community acceptability and buy-in. Agreement will thus need to be reached on referral pathways, facility-NGO-CHW relationships, mechanisms for inter-sectoral coordination and processes of community engagement and participation. This in turn will require a number of mind-set shifts. The appraisal found that many in the PHC system were supportive of closer links with CBS, but often did not appreciate its particular contribution to the health system and regarded it as a subordinate rather than independent sphere. As highlighted elsewhere [19], where the PHC system is not adequately inducted, professionals will naturally seek to draw community based cadres into health facilities as an “extra pair of hands”.

### Resourcing

Successful CHW programs are able to *“cultivate support and to withstand competition in the broader political and economic environment”* [11]*.* In many contexts, such support has occurred against a backdrop of a widely perceived human resource crisis [3]. In the Western Cape, the problem presents itself less as one of crisis than the need to elevate the status and improve the performance of an existing platform. While in the minds of many players the extent of health burdens and the need for better prevention and promotion justified reforms to CBS, the appraisal also encountered considerable scepticism of its capacity to deliver even its current mandate. Meeting the norms of Healthcare 2030 requires an expansion in numbers of CHWs and supervisors, a shift to full time employment and improved remuneration levels. In addition to better conditions of service for its core cadres, systems of financing, monitoring and evaluation and training, integrated into the routine functioning of PHC and district health systems, will be needed. In the face of multiple competing demands, a major challenge confronting CBS will be to garner evidence and sustained high-level political support to translate policy intent into concrete resource allocation.

### Governance

One of the key governance dilemmas in the Western Cape is whether to retain the NGO contracting system or not, especially since significant capacity for contract management has been established in the province. The DOH model (and the likely improvement in conditions of service) was seen by many as a way to stabilise community based services and achieve greater standardisation of approaches. The danger with this model is that the CHWs will be treated as workers on the lowest rung of the civil service, losing their community identity and increasingly drawn into facility based functions. As a number of case studies documented during the appraisal found, the NGO model has greater capacity for innovation and responsiveness, even if the transaction costs of managing contracts are high. As Pallas et al. [11] point out, *“CHW approaches are successful if they are at once strongly connected to the community and also have a clearly defined role and relationship with the formal health system”.* The added value of relative autonomy and community embeddedness may favour an NGO model, especially where there is some pre-existing capacity.

An NGO partnership system, however, requires capacity for managing contractual relationships that includes not only financial accounting and performance monitoring but also the trust relationships necessary for effective cooperation in a plural environment. The more decentralized these processes the more the possibility of establishing so-called “relational” contracting systems [24], between government and NGOs. The appraisal concluded that in the Western Cape, the NGO model did provide a basis for CBS, if relationships with PHC services were improved, and the sub-district played a more central role in the management of partnerships.

A strengthened community based system also entails coordinating actors who do not exist in formal hierarchical or contractual relationships with health services, such as providers from other sectors and community structures. Being able to build norms of responsiveness and answerability between these local players, despite the absence of formal lines of accountability is a key element of local governance of CBS. It requires the capacity to shift from modes of command-and-control (managing up and down) that are the dominant cultures within frontline service provision towards new relationships across organisational boundaries based on networking, cooperation and reciprocity (managing out). As with PHC players, this requires a mind-set shift at sub-district level, where the default approach will be to treat all relationships as hierarchical, or to avoid interactions that cannot be managed through command-and-control.

## Conclusions

The renewed focus on community based services as part of national health systems in South Africa, and the southern African region more generally, is due in part to the massive opportunities offered by the mobilisations around HIV/AIDS over the last two decades. Many of the difficulties of formalising and strengthening community based services, located at the interface between households and the formal health system, are well known. However, this paper seeks to place these challenges in a rapidly evolving contemporary context, not only with respect to changing health needs, but also in the range and complexity of actors involved, and the need for new relationships of coordination and accountability in plural health systems.

Drawing on an adapted version of the WHO building blocks model (“hardware”), and combining this with an actor centred and relational (“software”) analysis, the paper also provides an approach for holistically evaluating this sub-system. Ultimately, the central and most complex challenges in strengthening CBS lie less in technical systems design, than in making the case for investment through advocacy and evidence, and forging new relationships and a new CBS identity on the back of an established system. This requires considerable “managerial flexibility and strategic flair” and an ability to engage multiple actors, interest groups and organisations in a sustained fashion over time [25].

## Additional file

Additional file 1:
**Table S1.** Overview of appraisal findings. (DOCX 98 kb)

## Abbreviations

CBS: Community based services
CHW: Community health worker
DOH: Department of Health
PHC: Primary health care
