# Supplementary material for: The challenges of reshaping disease specific and care oriented community based services towards comprehensive goals: a situation appraisal in the Western Cape Province, South Africa
Source: BMC Health Serv Res. 2015 Sep 30;15:436. doi: 10.1186/s12913-015-1109-4 (PMC4589097; doi:10.1186/s12913-015-1109-4)
Supplement: Additional file 1: — Table S1. Overview of appraisal findings. (DOCX 98 kb) [file 12913_2015_1109_MOESM1_ESM.docx]

Additional file 1: Table S1: Overview of appraisal findings

| **Systems dimension** | | **Findings** | **Challenges & Recommendations** | **Source of data** |
| --- | --- | --- | --- | --- |
| Outputs | Access | Well established system with 0.78 CHWs/1000 population;  28% shortfall in numbers of CHWs to meet projected norms of Healthcare 2030; and expansion of working hours from half day to full time required;  Uneven distribution of CHWs by district. | Expand infrastructure;  Ensure geographical equity. | Routine data |
|  | Quality | High levels of community acceptability, although expectations less than for professionals;  Observed technical quality variable and dependent on supervisor involvement, NGO leadership and link to local facility;  Absence of quality audits. | Improved resources for and systems of supervision;  Develop quality assurance systems. | Community focus group discussions  Observations  KI Interviews |
|  | Patient-centredness | Restricted to a limited number of care routines;  CHWs follow instructions, rather than exercise discretion | Build CHW capacity to assess household needs, make judgements and individualise responses. | Observations |
| Service delivery | Roles | Roles include: home based care for referrals from hospitals and other parts of the health system (most significant role); community based drug distribution for chronic non-communicable diseases (so-called “CDL” clubs); adherence support and counselling for HIV/TB; school health services; and participation in periodic “four seasons” (community health promotion) campaigns;  In urban areas, provided as separate single purpose activities, through separate teams/NGOs, in rural areas integrated;  Limited preventive and promotive roles; only one NGO provides integrated management of childhood illness in province;  Missed opportunities at household and community levels. | Consensus on priority roles and core package;  Shift to greater preventive and promotive focus;  Shift to comprehensive approach within teams, with greater focus on MCH. | NGO databases  Observations  KI interviews |
|  | Delivery model | CHWs employed by NGOs who are contracted by district authorities;  Major emphasis is on providing “dehospitalised” care services in the home and on elderly;  Reactive and referral rather than proactive, population oriented approach;  Activities not coordinated in geographical areas and with PHC/sub-district system. | Institute a proactive population based approach;  Combine household with community based roles;  Coordinate activities with focus on catchment areas of local PHC facilities. | Routine data  Observations  Focus group discussions |
|  | Teams | In rural areas a generalised “home based CHW”, in urban areas, a mix of home based CHWs with specialised workers focusing on NCDs, adherence supporter for HIV/TB; and school health services;  Ratio of 1 supervisor: 20 CHWs; limited direct household supervision. | Integrate functions within geographically defined areas;  Increase number of supervisors (ratio 1:10 workers proposed in Healthcare 2030). | NGO databases |
|  | Referral | Referral from hospitals via sub-district and NGO;  Limited links with PHC facilities. | Build primary relationships (referral, support and accountability) with local primary health care facilities. | KI Interviews |
| Resources & support | Human resource: retention, remuneration;  Training | High turnover of CHWs around a stable core; remuneration too low to ensure retention;  51% of CHWs at entry level (untrained);  Nationally accredited training but focused on limited care roles; poor alignment with needs; and between classroom and practical learning;  Orientation/induction training systems mostly non-existent. | Improve remuneration and ensure retention;  Review training systems, better alignment with needs, and including basic, in-service and orientation training. | Routine data  NGO databases  KI interviews  Document reviews |
|  | M&E systems | Large amounts of data collected and submitted on household activity but considered poor quality and not trusted or used by managers;  Activity indicators poorly defined and difficulty of fitting this with variable NGO activity;  No output and outcome indicators;  System poorly integrated into district health information systems | Develop M&E systems integrated into routine district health information system;  Ensure reliable reporting on activity and link this to outcomes;  Make use of new technologies (mHealth). | KI Interviews  Review of documents/  Registers |
|  | Financing & infrastructure | Resource allocation from special HIV and other grants, not integrated into core budget processes;  Good financial accountability, but lack of trust in value for money (performance accountability) from NGO contracts;  Inadequate remuneration of CHWs;  Poor resourcing of transport, communication (mobile phones) and equipment. | Build M&E and other systems of accountability;  Increase resourcing;  Provide evidence on value for money;  Integrate budgets and resource allocation into DHS. | KI Interviews  Observations  Focus group discussions |
| Governance & Management | NGO contracting | NGOs source of innovation but activities not standardised;  Contracting at district level, through vertical community based services directorates with little involvement of sub-district managers;  Separate from governance of PHC system and seen as outside the control of the sub-district. | Develop provincial guidance and support for NGOs on core package, M&E, training;  Build sub-district capacity for priority setting, planning and relational NGO contracting;  Integrate management of community and facility based PHC services. | KI Interviews  Observations |
|  | NGO/CHW/  PHC links | Coordination and relationships with PHC system uneven and poorly developed;  Lack of processes of local inter-sectoral action, coordination with local government (environment health), or community accountability. | Develop local governance of community based services in PHC catchment areas;  Build mechanisms for inter-sectoral action. | KI Interviews  Observations  Focus group discussions |
